# The effectiveness of educational practice in diabetic foot: a view from Brazil

**DOI:** 10.1186/1758-5996-2-45

**Published:** 2010-06-29

**Authors:** Maria I Anselmo, Marcia Nery, Maria CR Parisi

**Affiliations:** 1 Endocrinology Department, Medical School of the State University of Sao Paulo, Sao Paulo, Brazil

## Abstract

**Background:**

The aim of the present study was to evaluate the prevention and self-inspection behavior of diabetic subjects with foot at ulcer risk, no previous episode, who participated in the routine visits and standardized education provided by the service and who received prescribed footwear. This evaluation was carried out using a questionnaire scoring from 0-10 (high scores reflect worse practice compliance).

**Results:**

60 patients were studied (30 of each sex); mean age was 62 years, mean duration of the disease was 17 years. As for compliance, 90% showed a total score ≤5, only 8.7% regularly wore the footwear supplied; self foot inspection 65%, 28,3% with additional familiar inspection; creaming 77%; proper washing and drying 88%; proper cutting of toe nails 83%; no cuticle cutting 83%; routine shoe inspection 77%; no use of pumice stones or similar abrasive 70%; no barefoot walking 95%.

**Conclusion:**

the planned and multidisciplinary educational approach enabled high compliance of the ulcer prevention care needed in diabetic patients at risk for complications. In contrast, compliance observed for the use of footwear provided was extremely low, demonstrating that the issue of its acceptability should be further and carefully addressed. In countries of such vast dimensions as Brazil multidisciplinary educational approaches can and should be performed by the services providing care for patients with foot at risk for complications according to the reality of local scenarios. Furthermore, every educational program should assess the learning, results obtained and efficacy in the target population by use of an adequate evaluation system.

## Background

The presence of foot ulcerations in diabetic individuals at risk for complications is a frequent event, with an estimated 15% of all diabetic individuals experiencing an episode of ulceration at some point during their life. Although most of the ulcerations heal, 70% of the cases recur, frequently progressing to unavoidable limb amputation. Several factors are involved in the development of this process which include Peripheral Neuropathy (PN), Peripheral Vascular Disease (PVD), limited joint mobility and repeated trauma from abnormal load distribution on the foot [1,2]. However, this unfavorable progression can be modified provided effective preventive measures are adopted such as adequate guidance regarding: 1- lack of sensitivity and/or presence of peripheral vascular disease and its implications, 2- hygiene and moisturizing practices, 3- use of adequate footwear (when indicated), 4- mandatory daily self-care (self-examination).

These guidelines should be extended to family members and caregivers as many diabetic patients at risk for complications such as obesity are frequently impaired for fulfilling self-examination.

Therefore, preventive and care practices should provide guidance on the correct way to wash, dry and moisturize the feet as well as on how to cut nails and not trim cuticles, or use pumice stones and similar abrasive objects, use appropriate footwear, perform regular foot and footwear inspection and never walk barefoot [3-5].

The aim of the present study was to evaluate the efficacy of an educational practice routinely used at the service and developed to guide diabetic patients at risk for complications.

## Patients and Methods

This was a cross sectional study. Sixty consecutive outpatients under treatment at the service and who participated in all routine care and educational guidance, classified according to the criteria from the International Working Group on Diabetic Foot Classification System [6] were studied.

Target population was followed-up for at least two years, had participated in the complete treatment program and for longer than one year in the educational program, besides periodically attending medical visits.

Presence of Neuropathy, Peripheral Vascular Disease and feet deformities were specifically assessed. Neuropathy diagnosis was determined using vibration perception (128 Hz tuning fork) at two sites (hallux pulp and malleolus), point pressure (Semmes-Weinstein 10 g monofilament) at seven sites, and ankle reflexes [6]. Arterial blood supply to the foot was determined by palpation of the dorsalis and posterior tibial foot pulses. For diminished or impalpable pulses ankle brachial pressure index (ABPI) was performed. The presence of callosities, toe deformities (i.e. claw, hallux valgus) or other signs of plantar overload were considered risk deformities.

The full program comprises three steps: 1- Medical visit and examination with diagnosis and detailed explanation of the disease, risk of ulcer progression, risk of amputation and footwear prescription (when indicated); 2- individual visit with a nursing professional directed towards general preventive care and review of the medical prescriptions (15 minutes long in average); 3- educational group, set up by the nursing team, directed to patients and family members and/or caregivers (with the objective of reinforcing self-examination instructions and in average 45 minutes long).

In order to assess the efficacy of the program, daily routine of foot self-examination was analyzed using a simple 10-item questionnaire in which the following parameters where assessed: self foot inspection, additional foot inspection performed by family member, adequate washing and drying, creaming, toe-nail and cuticle cutting, use of proper footwear, routine shoe inspection, no use of pumice stones or similar abrasive objects, no barefoot walking. Each question was awarded a "0/1" score according to the reply, where "0" meant a correct procedure and "1", inadequate. Each patient had a final 0-10 score for the questionnaire, where high scores represented an increased number of inadequate daily practices.

Statistical analysis was performed using SAS statistical software (SAS Institute Inc., Cary, NC) and a *p *value of 0.05 was considered statistically significant.

## Results

Of the 60 studied patients, 30 were male, 30 female. Mean age was 62 years; mean duration of the disease was 17 years (baseline characteristics of studied subjects - Table 1).

**Table 1:** Baseline characteristics of studied subjects

Subjects (*n*60)	Percentage *n *(%)
Male	30 (50%)
Female	30 (50%)
Age (years)	61, 4 (32-82)
Duration of diabetes (years)	16.95 6 (2-41)
	
Smoking	4 (6.6%)
	
Diabetic retinopathy	43 (71.6%)
Diabetic nephropathy	37 (61.6%)
Hypertension	50 (83.3%)
Cardiovascular disease	30 (50%)
Distal sensory neuropathy	60 (100%)
Peripheral vascular disease	13 (21,6%)

Patients' performance was: 77% adequate moisturizing, 88% proper washing and drying, 83% proper toe-nail cutting, 83% no cuticle trimming, 77% routine shoe inspection, 70% no use of pumice stones or similar abrasive objects, 95% no barefoot walking, but only 5 patients (8.7%) regularly wore the provided footwear.

There were no score differences observed among the sexes (Table 2). Until the conclusion of this paper, there was no ulcer in the studied population.

**Table 2:** Patient Score Distribution by Sex

Sex	Score	Frequence	Percentage %
Female	1	6	20.0
	2	5	16.7
	3	8	26.7
	4	6	20.0
	5	2	6.7
	6	3	10.0
	Total	30	100.0
	
Male	1	3	10.0
	2	12	40.0
	3	9	30.0
	4	1	3.3
	5	2	6.7
	6	1	3.3
	7	2	6.7
	Total	30	100.0

## Discussion

The results obtained demonstrate that the multidisciplinary educational program conducted led to constructive attitudes in self-examination. This was previously described by other groups which observed that well-integrated multidisciplinary teams are associated with better clinical outcome [7-10].

It was a striking surprise to verify that 90% of the patients performed all suggested measures; and also that less than 10% of the patients made use of the provided footwear. Although this study was not designed to evaluate compliance of footwear use the finding is important and similar to results published by other authors from reference centers of great expertise in the field [11].

Unfortunately, our evaluation did not focus on questions regarding the poor compliance in use of the footwear. However, critically analyzing the prescribed footwear, which was custom made according to established standards for safe and adequate footwear [12], it was observed that they were far from attractive. Probably the esthetic aspect played a relevant role in the lack of compliance, as has previously been observed [13,14].

In 1994, one of the first studies to evaluate footwear use in patients with severe neuropathy and history of foot ulcerations concluded that differences in age, perception of foot abnormalities and health status, as well as other distressing medical conditions (i.e. renal replacement therapy, previous minor amputations), in addition to cosmetic reasons, may affect the patients' compliance [15].

The esthetic aspect seems to be so important that 10 years later an Editorial states: "Whereas bad shoes cause ulcers and "ugly" shoes are likely to remain in the closet, a major effort is required to demonstrate that the good shoes do actually benefit our high-risk patients" [16].

Another possible reason for such low compliance may be related to what extent the multidisciplinary team routinely and effectively practices footwear prescription. Footwear prescription for diabetic patients at risk for complications is a controversial topic where even the proposed guidelines often leave gaps not addressed. Depending on the healthcare service, most of what is prescribed is based on empirical opinions [17,18].

It therefore seems appropriate, under such considerations, and as per Reiber et al [19], to defend those healthcare professionals should guide patients on identifying footwear characteristics and on choosing footwear adequately. Instruction on footwear characteristics does in fact provide significant information about foot protection and increase in ulceration risk, better enabling patients to choose from available footwear and also recognizing hazardous footwear.

Careful review of the footwear provided at our service and further new analysis of its acceptability are most required.

Finally, we believe that it would be interesting to report this local experience considering that in Brazil educational initiatives involving diabetic patients at risk for complications are still limited. The continental dimensions of our country and social-economic differences among the regions cannot be forgotten. This experience is feasible at centers where family healthcare programs have not yet been implemented as both medical and nursing professionals provide services at any center from the National Health System (SUS-Brazil). In this context, it is our belief that every educational program should be carried out with systematic evaluation of the learning process, results and efficacy in the target population.

## Conclusion

A planned and multidisciplinary educational approach enabled high compliance of ulcer prevention care needed in patients at risk for diabetic foot complications. However, compliance for use of provided footwear was extremely low and for reasons only partially understood. Careful review of the footwear provided at our service and further new analysis of its acceptability are most required.

In countries of such vast dimensions as Brazil multidisciplinary educational approaches can and should be performed by the services providing care for diabetic patients at risk for complications, respecting the reality of local scenarios. Furthermore, every educational program should assess the learning, results and efficacy in the target population with an adequate evaluation system.

## Competing interests

The authors declare that they have no competing interests.

## Authors' contributions

MIA applied the questionnaires used in this paper, collected the used data and participated in the results analyses. MN participated in the design of the study and participated in the drafted the manuscript. MCRP conceived of the study, participated in its coordination and participated in the drafted the manuscript. All authors read and approved the final manuscript.
